# ADDRESSING SOCIAL ISOLATION IN NURSING HOMES DURING COVID-19: A NATIONAL SURVEY

**DOI:** 10.1093/geroni/igac059.2967

**Published:** 2022-12-20

**Authors:** Natalia Nielsen, J Lee Hargraves, Carol Cosenza, Emily Lim, Adrita Barooah, Emily McPhillips, Catherine Dube, Kate Lapane

**Affiliations:** University of Massachusetts Medical School, Worcester, Massachusetts, United States; University of Massachusetts - Boston, Boston, Massachusetts, United States; University of Massachusetts Boston, Boston, Massachusetts, United States; University of Massachusetts Boston, Boston, Massachusetts, United States; University of Massachusetts Boston, Boston, Massachusetts, United States; UMass Chan Medical School, Worcester, Massachusetts, United States; UMass Chan Medical School, Worcester, Massachusetts, United States; University of Massachusetts Chan Medical School, Worcester, Massachusetts, United States

## Abstract

COVID-19 related policies introduced extraordinary social disruption in nursing homes. In response to the unprecedented COVID-19 pandemic, congregated long term care living facilities attempted and/or implemented innovative intervention strategies to alleviate loneliness in residents. We surveyed Directors of Nursing/Administrators of 1,669 homes sampled in strata defined by size (number of beds 30–99, 100+) and quality ratings (1, 2–4, 5) between February-May 2022. The response rate was 30%. Almost 2/3rds of respondents completed it online and the rest via paper. Analyses included nonresponse survey weights to provide nationally representative results. Among a list of 17 situations that occurred, staff shortages was identified as extremely stressful by the majority. Staff were extremely stressed about doing more to meet resident needs and keeping up with rapidly changing regulations which often lacked clinical sense. One third of respondents were extremely concerned about their home’s ability to meet residents’ social needs before vaccines, dropping to 13% after vaccines. Nursing homes tried and perceived as most useful using technology (tablets, phones, emails), assigning staff as a family contact, and staff spending more time with residents. Nearly 60% were extremely concerned about staff burnout/mental health before vaccines and 40% remained extremely concerned after vaccines. Many nursing homes attempted to mitigate the harmful effects of social isolation during the pandemic, despite the stressful circumstances in which staff worked. The extent to which various approaches were implemented varied. While concerns about social isolation reduced after vaccines were available, administrators remain extremely concerned about staff burnout and mental health.

